# Revising the distribution of a threatened goby, *Apocryptodon punctatus* (Perciformes, Oxudercidae), in Japan with the discovery of an isolated population

**DOI:** 10.3897/zookeys.645.10755

**Published:** 2017-01-12

**Authors:** Atsunobu Murase, Ryutei Inui, Ryohei Miki, Yusuke Miyazaki

**Affiliations:** 1 Nobeoka Marine Science Station, Faculty of Agriculture, University of Miyazaki, 376-6 Akamizu, Nobeoka, Miyazaki 889-0517, Japan; 2 Department of Marine Biology and Environmental Sciences, Faculty of Agriculture, University of Miyazaki, 1-1 Gakuen-Kibanadai-Nishi, Miyazaki 889-2191, Japan; 3 Graduate School of Sciences and Technology for Innovation, Yamaguchi University, 2-16-1 Tokiwadai, Ube, Yamaguchi 755-8611, Japan; 4 Interdisciplinary Graduate School of Agriculture and Engineering, University of Miyazaki, 1-1 Gakuen-kibanadai-nishi, Miyazaki 889-2192, Japan; 5 Department of Child Education and Welfare, Shiraume Gakuen College, 1-830 Ogawa-cho, Kodaira-shi, Tokyo 187-8570

**Keywords:** Endangered species, estuarine fish, Gobioidei, Red List, tidal flat, voucher

## Abstract

Five specimens of a threatened goby, *Apocryptodon punctatus* (21.2–40.1 mm in standard length), were collected at a mudflat site of Kushima City, Miyazaki Prefecture, Kyushu, southern Japan over two seasons, autumn (September 2015) and spring (April 2016). A review of distributional records of *Apocryptodon punctatus* revealed that this population represents the southernmost record of the species in Japanese waters, and is isolated *ca.* 200 km south-southwest from the nearest point of the main range of the species along the Pacific coast of Japan. Publicising this population will help conserve it and its vulnerable habitat.

## Introduction

*Apocryptodon punctatus* Tomiyama, 1934, an estuarine gobiid fish of the subfamily Oxudercinae (Murdy 1989), now the family Oxudercidae (Nelson et al. 2016), is known from central to western Japan (Inui 2015), the western coast of South Korea (Kim et al. 1986, Youn 2002) and Taiwan (Chen and Fang 1999). This species inhabits silty mudflat environments utilizing the burrows of alpheid shrimps (Dôtu 1961; Koyama et al. 2017). It is an ecological indicator for natural tidal flat environments (Suzuki 2003) and recent ecological studies have shown habitat preferences and symbiotic partner specificity for the species (Koyama et al. 2016, 2017). Although the species is considered threatened because of the vulnerability of its habitat (Iwata 1997, Suzuki 2003), recent increases in field sampling efforts have resulted in the discovery of several new locality records (Inui 2015). Consequently, the species’ conservation status in the Red Data Book for wildlife of Japan has dropped from “Endangered (EN)” in 2007 to “Vulnerable (VU)” in 2015. However, this does not reflect an improvement in the species’ habitat, and habitat degradation remains a concern (Inui 2015). Sato and Aizawa (1992) summarized literature and specimen records of the species at that time, including a distribution map of the species (with 13 points on the map); the distribution showed a belt-like shape from central to western Japan with sparse distribution in central Japan. Of these records, the southernmost distributional point (Nichinan City, southern part of Miyazaki Prefecture) was clearly separated from the main distribution, ca 200 km to the south. However, this record was based on a juvenile specimen (Masahiro Aizawa personal communication) and there has been no further record from Miyazaki Prefecture until very recently (Inui 2015). Given the lack of known populations in the Miyazaki Prefecture, the origin of the Nichinan City specimen was uncertain. In recent faunal diversity surveys of estuaries along the coasts of Miyazaki Prefecture, the authors collected several specimens of *Apocryptodon punctatus* from Kushima City, located 20 km southwest of Nichinan City. Distributional records of threatened species and their publicity are important contributors to policy decisions regarding the conservation of vulnerable species and their habitats (Inui and Koyama 2014). Furthermore, unusual occurrences of a species may indicate a biodiversity hotspot or other unusual conditions (Hiscock 2014). This note describes the specimens of *Apocryptodon punctatus* from Kushima City, and reports on the habitat in order to elucidate the population status at this site. Additionally, in order to understand the range of *Apocryptodon punctatus* in Japan, recent records of the species were reviewed, including unpublished specimen data that supported distributional information for each prefecture in Inui (2015).

## Materials and methods

**Figure 1.**
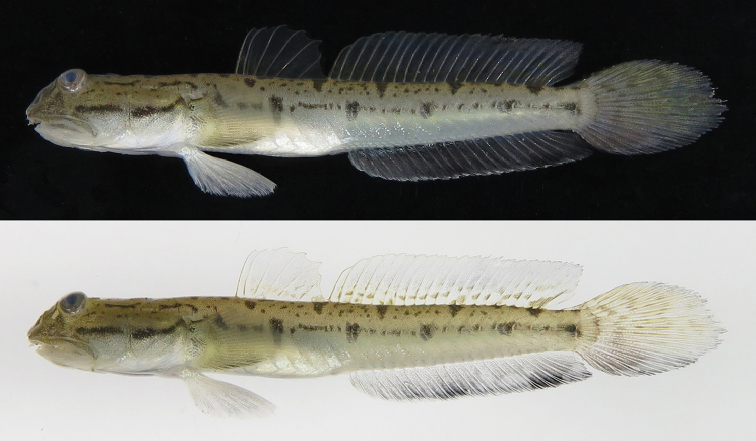
Lateral view of a fresh specimen of *Apocryptodon punctatus*, KPM-NI 40559, 35.6 mm SL, collected from a mudflat estuary of Kushima City, Miyazaki Prefecture, Kyushu, Southern Japan. Top, photo number KPM-NR 166540B; bottom, photo number KPM-NR 166540A.

Specimens of *Apocryptodon punctatus* were collected using hand nets from a mudflat estuary of Kushima City, the southernmost part of Miyazaki Prefecture, Kyushu, southern Japan (detailed information of the locality omitted for conservation purposes), in the Japanese autumn (= end of summer, September 27, 2015) and spring (April 8, 2016). The specimens were immediately killed by placing them in a mixture of environmental water and ice in a plastic bag. Thereafter, the fishes were fixed in 10% formalin and subsequently preserved in 70% ethanol. Color photographs when fresh (Fig. 1) were taken after approximately 30 minutes in fixation. All specimens and photos were deposited in the ichthyological collection and image database of the Kanagawa Prefectural Museum of Natural History (KPM-NI for specimens, KPM-NR for photos). The following five specimens were examined (photo numbers in parentheses): KPM-NI 40542 (KPM-NR 166524), 26.6 mm SL (standard length), 27 Sep. 2015, collected by A. Murase and Y. Miyazaki; KPM-NI 40543 (KPM-NR 166525), 21.2 mm SL, same data as KPM-NI 40542; KPM-NI 40558 (KPM-NR 166539), 28.9 mm SL, 8 April 2016, collected by A. Murase and R. Miki; KPM-NI 40559 (KPM-NR 166540), 35.6 mm SL, same data as KPM-NI 40558; KPM-NI 40560 (KPM-NR 166541), 40.1 mm SL, same data as KPM-NI 40558.

Counts and P-V relation (i.e., the relationship between pterygiophores of the dorsal fins and vertebrae) followed Akihito (1984), with vertebrae data taken from radiographs. Caudal-fin rays included those on hypural plates. Measurements were made with needle-point calipers to the nearest 0.1 mm, according to Hubbs and Lagler (2004) except for the following: body depth, vertical distance from anal-fin origin to second dorsal-fin base; prepelvic length, distance from snout tip to anterior margin of base of pelvic-fin spine; preanal length, distance from snout tip to anal-fin origin; caudal peduncle depth, depth at vertical trough point of attachment of last anal-fin membrane to caudal peduncle; pectoral-fin length, length of longest ray; pelvic-fin length, distance from base of pelvic-fin spine to posteriormost extremity of fin.

In order to elucidate the condition of the environment, salinity and water temperature in tidal pools on the mudflat where the specimens of *Apocryptodon punctatus* were captured were measured at the time of fish sampling using YK-31SA (Sato Shouji Inc., Kawasaki) and TT-508 (Tanita, Tokyo). In addition, the percentages of silt and clay were calculated following Koyama et al. (2016a) using a sediment core of 2.5 cm depth and 5 cm diameter. These water environment and sediment samplings were repeated five times and 20 times on each collection date (27 September 2015 and 8 April 2016) respectively.

Inui (2015) updated the distribution of *Apocryptodon punctatus* in Japanese waters and listed the names of prefectures where the species has been recorded. While several new prefecture records were included, Inui (2015) provided no specimen data supporting the records. In order to accurately document the known distribution of *Apocryptodon punctatus*, a list of localities of the species was summarized on the basis of published literature and the second author’s unpublished specimen data that had been used for the distributional update in Inui (2015). Those specimens were deposited in the Tokushima Prefectural Museum (TKPM-P). Of the prefectures listed as localities of *Apocryptodon punctatus* by Inui (2015), Mie and Kagoshima Prefectures have no museum specimen records (the collected specimens are either uncatalogued or missing); locality details in those prefectures follow Ryutei Inui’s unpublished data.

## Results

### Description

Counts and measurements of *Apocryptodon punctatus* collected from Kushima City are shown in Table 1. P-V relation 3/I II II I 0/9 (based on only the three larger specimens due to the obscurity of pterygiophores in the smaller specimens). A supraorbital pore present just behind eye.

Body elongate and more compressed posteriorly. Eyes small and prominent dorsally. Mouth large, horizontal and its posterior edge positioned behind a vertical line from posterior edge of eye. First and second dorsal fins close and connected by small membrane.

**Table 1.** Counts and proportional measurements of *Apocryptodon punctatus* (*n* = 5) from Kushima City, Miyazaki Prefecture, Kyushu, southern Japan.

Standard length (mm)	21.2–40.1
**Counts**	
Dorsal-fin rays	VI-I, 22
Anal-fin rays	22 or 23
Pectoral-fin rays	22 or 23
Pelvic-fin rays	I, 5
Caudal-fin rays (upper + lower)	7 + 6
Vertebrae (precaudal + caudal)	10 + 26
**In % of standard length**	
Total length	125.6–128.7
Head length	29.2–31.2
Snout length	7.9–9.3
Upper-jaw length	15.2–16.0
Interorbital width	0.2–0.5
Orbit diameter	5.2–6.4
Body depth	12.6–14.0
Predorsal length	38.5–40.1
Prepelvic-fin length	29.2–30.1
Preanal-fin length	58.5–60.8
Caudal-peduncle length	4.8–5.7
Caudal-peduncle depth	7.6–8.2
Length of 1^st^ dorsal-fin base	15.6–17.0
Length of 2^nd^ dorsal-fin base	40.8–41.4
Length of anal-fin base	36.5–38.8
Pectoral-fin length	16.0–17.5
Pelvic-fin length	17.5–19.3
Length of 1^st^ spine of 1st dorsal fin	9.7–10.4
Length of 2^nd^ spine of 1st dorsal fin	10.7–11.3
Length of 1^st^ spine of 2^nd^ dorsal fin	7.7–9.0
Length of 1^st^ soft-ray of 2^nd^ dorsal fin	9.5–11.1
Length of 1^st^ anal-fin soft-ray	5.2–5.9
Length of 2^nd^ anal-fin soft-ray	6.9–7.5

Head and body dark yellow dorsally, lower part of head and trunk white, tail greyish white ventrally. Bold black bar horizontally across centre of preopercular and opercula (bar posteriorly oblique reaching to posterodorsal edge of operculum in larger specimens). Dark bar on occipital region across dorsal edge (bar shape differs between individuals, being a simple bar, fine arch, or eyeglass-like spot). Two dark bars present across dorsal edge of nape, laterally appearing as dark spots. Dark bar (may be wedge like) across anterior part of 1^st^ dorsal-fin base; three dark bars across region from end of 1^st^ dorsal-fin base to centre of 2^nd^ dorsal-fin base. Blotch present across posterior base of 2^nd^ dorsal fin. These bars or blotch on dorsal edge appearing as a simple spot, line or saddle-like spot in lateral view. Dark blotch or spot present on upper anteriormost part of region beneath pectoral-fin (absent in KPM-NI 40542). Five small dark blotches (two anteriormost vertically oblong in shape and latter three shorter or circular) present on body axis from trunk to caudal-fin base, connected to each other with dark horizontal lines. Dorsal-fin rays dark yellow. First dorsal-fin entirely transparent but 2^nd^ dorsal-fin slightly darker posterodorsally with numerous horizontal dark yellow spots on lower part of fin. Anal-fin rays nearly transparent, whitish. Lower edge of anal fin with white margin, becoming broader anteriorly in larger specimens; area above white region blackish, darker posterodorsally (darker area separated into two separate wide blotches in KPM-NI 40559: Fig. 1). Caudal-fin rays nearly transparent; upper and central area of fin dark yellow, lower part blackish. Pectoral fin nearly transparent, dark yellow; lower part of fin blackish with white lower margin. Pelvic fin entirely whitish with nearly transparent membrane.

### Habitat

The Kushima City specimens of *Apocryptodon punctatus* were captured on a small mudflat zone (ca 200 m^2^) that at low tide reveals a small stream flowing into the mouth of a wider river. The mean salinity level of the location was 2.7 ± 0.1 (± SD, ranging 2.6–2.9) and 2.3 ± 0.1 (2.1–2.5) in autumn and spring respectively. It is considered a polyhaline environment (sensu McLusky and Elliott 2004) over the two seasons, while the nearby stream had zero salinity at the sampling time. Mean water temperature was 29.6 ± 1.6°C (27.8–31.5°C) and 21.8 ± 0.5°C (21.4–22.5°C) in autumn and spring respectively. The mean ratio of silt and clay in the location was 31.7 ± 7.5% (17.7–45.8%) and 35.7 ± 6.3% (19.4–45.1%) in autumn and spring respectively. The mean ratio of sand (66.0 ± 1.5% and 59.5 ± 5.2% in autumn and spring respectively) was greater than that of silt and clay, and that of gravel (2.3 ± 1.5% and 4.8 ± 1.5% in autumn and spring respectively) was clearly less than that of silt and clay showing that the composition of sediments in the location was mainly sand, with some silt and clay and almost no gravel.

### Distribution

**Figure 2.**
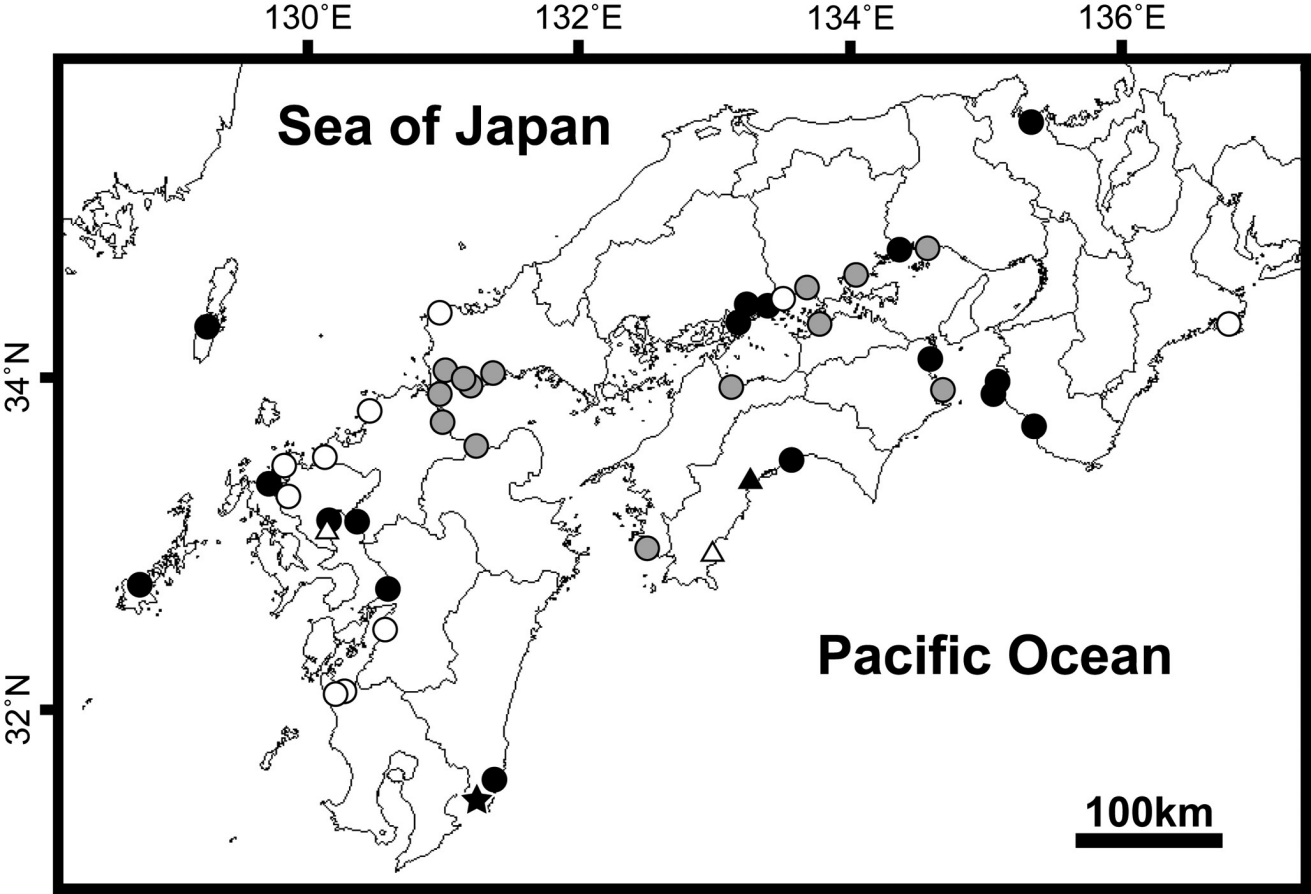
Distribution map of *Apocryptodon punctatus* in Japanese waters. Solid star indicates the present record from Kushima City, Miyazaki Prefecture, Kyushu; solid circles, records with voucher specimens deposited in a museum; gray circle, records with additional museum specimens in the present study; open circles, records with uncatalogued specimens; solid triangle, photo record; open triangles, literature records without a voucher.

The distribution records of *Apocryptodon punctatus* are summarized in Table 2 and each locality is mapped in Fig. 2 (45 localities).

**Table 2.** Distribution records of *Apocryptodon punctatus* from Japanese waters based on literature and voucher sources.

Locality			Source	
Region	Prefecture	City, town or island	Literature	Voucher
Pacfic coast (including Seto Inland Sea)	Mie	Watarai-gun	Inui (2015)	US
	Wakayama	Kainan City	Senou and Kitamura (1982)	MS
		Arida City	Kishino and Nomoto (2000)	MS
		Tanabe City	Suzuki and Wada (1999)	MS
	Tokushima	Tokushima City	Sato and Aizawa (1992)	MS
		Anan City	Present study	adMS
	Kochi	Kochi City	Miyake et al. (2006)	MS
		Suzaki City	Okamura (2002)	P
		Shimanto City	Okamura (2002)	Non
	Miyazaki	Nichinan City	Sato and Aizawa (1992)	MS
		Kushima City	Present study	adMS
Seto Inland Sea	Hyogo	Tatsuno City	Present study	adMS
		Ako City	Suzuki and Masuda (1993)	MS
	Okayama	Okayama City	Present study	adMS
		Kurashiki City	Present study	adMS
		Kasaoka City	Dôtu (1961)	US
	Hiroshima	Fukuyama City (east)	Yoshigou and Nakamura (2002)	MS
		Fukuyama City (west)	Yoshigou (2001)	MS
		Innoshima City	Yoshigou (2001)	MS
	Yamaguchi	Yamaguchi City	Present study	adMS
		Ube City	Present study	adMS
		Sanyo-onoda City	Present study	adMS
		Shimonoseki City	Present study	adMS
	Kagawa	Marugame City	Present study	adMS
	Ehime	Saijyo City	Present study	adMS
		Ainan-cho Town	Present study	adMS
	Fukuoka	Kitakyushu City	Present study	adMS
		Yukuhashi City	Present study	adMS
	Oita	Nakatsu City	Present study	adMS
Sea of Japan and East China Sea	Kyoto	Maizuru City	Matsui et al. (2011)	MS
	Yamaguchi	Nagato City	Mori (1995)	US
	Fukuoka	Fukutsu City	Inui et al. (2012)	US
		Itoshima City	Inui et al. (2012)	US
		Yanagawa City	Tomiyama (1934)	Holotype
	Saga	Higashimatsuura-gun	Fujii and Asayama (2013)	US
		Imari City	Fujii and Asayama (2013)	P, US
		Kishima-gun	Sato and Aizawa (1992)	MS
		Kashima City	Dôtu (1961)	Non
	Nagasaki	Tsushima Island	Yoshigou and Nakamura (2003)	MS
		Matsuura City	Sato and Aizawa (1992)	MS
		Goto Islands	Yoshigou and Nakamura (2003)	MS
	Kumamoto	Uto City	Sato and Aizawa (1992)	MS
		Yatsushiro City	Koyama et al. (2016a)	US
	Kagoshima	Izumi City	Inui (2015)	US
		Akune City	Inui (2015)	US

Voucher: adMS , additional museum specimens in the present study ; MS , museum specimens ; P , photo ; US , uncataloged specimens .

## Discussion

The five specimens collected from Kushima City corresponded well with the earlier descriptions of *Apocryptodon punctatus* in Tomiyama (1934), Murdy (1989), Sato and Aizawa (1992) and Akihito et al. (2013) in having the following diagnostic characters: dorsal-fin element VI-I, 22; mouth large, its posterior edge clearly positioned behind posterior edge of eye; five small dark blotches (two anteriormost vertical, oblong shape) present on body axis from trunk to caudal-fin base, connected by dark horizontal lines. The blotches on the lateral body of the Kushima City specimens differ somewhat with the descriptions in Tomiyama (1934) and Murdy (1989) in having circular blotches on the posterior part of the body. These earlier authors observed larger specimens (40–80 mm in total length, TL, in the former, 49–67 mm SL in the latter) for their description whilst the specimens in the present study ranged from 21.2–40.1 mm SL. The observed coloration differences may be ontogenetic or geographic variation, as mentioned in Matsui et al. (2011).

The first comprehensive review of distributional records of *Apocryptodon punctatus* since Sato and Aizawa (1992) resulted in many more localities than the 13 of Sato and Aizawa (1992), and included the northernmost (Maizuru Bay, Kyoto) and easternmost (Watarai-gun, Mie) records. The primary distribution of *Apocryptodon punctatus* is across mainland Honshu and the northwestern part of Kyushu, appearing as a diagonal band through western Japan (Fig. 2). This distributional review also revealed that the specimens from Kushima City, Miyazaki Prefecture, represent the southernmost records within Japanese waters, being ca 200 km south-southwest of Ainan-cho, Ehime Prefecture, the closest point on the Pacific coast (Fig. 2, Table 2). The authors’ recent survey of fish fauna in thirty estuaries along 100 km of the coast of Miyazaki Prefecture found *Apocryptodon punctatus* in the estuary of Kushima City only (Atsunobu Murase unpublished data). The record from Nichinan City (Sato and Aizawa 1992) was based on a single juvenile specimen (LIAIP1985-325, collected on 4 Oct. 1985). In addition, there have been no further records of the species and, in recent years, the preferred habitat of *Apocryptodon punctatus* in the city has been largely lost (Masahiro Aizawa, personal communication). On the other hand, several specimens have been captured over two seasons (autumn and spring) and other individuals observed (Atsunobu Murase unpublished data) on the mudflat estuary of Kushima City, located ca. 20 km south of Nichinan City. This mudflat has a recorded salinity of 2.1–2.9 at low tide, maintaining a higher salinity level than an adjacent stream (zero salinity), and is composed of a maximum of more than 45% silt and clay (on average more than 35% and 31% in spring and autumn respectively). The occurrence rate of *Apocryptodon punctatus* reaches its maximum at a silt and clay level of around 60%, in the estuary of Kuma-gawa River, western Kyushu (Koyama et al. 2016, Akihiko Koyama, pers. comm.). The silt and clay levels recorded in this study are approaching the level recorded in that previous study. Furthermore, several unidentified alpheid shrimps that may be symbiotic partners for *Apocryptodon punctatus* (Dôtu 1961, Koyama et al. 2017) have been observed in this mudflat (Atsunobu Murase unpublished data). *Apocryptodon punctatus* matures at 60 mm TL in Ariake Bay, western Kyushu (Dôtu 1961), but the maximum size recorded in the present study was 50.5 mm TL (KPM-NI 40560). Temperate fish species often have a smaller size at maturity in lower latitudes (i.e., warmer conditions) than higher latitudes (Kuriiwa et al. 2014, Trip et al. 2014, Stocks et al. 2015). Kushima City is located more than 100 km south-southeast of Ariake Bay and has warmer conditions in the coastal zone given its proximity to the warm Kuroshio Current (mean surface water temperature in February ca. 19°C in the former vs ca 14°C in the latter: Japan Oceanographic Data Center 2016); this latitudinal size variation could therefore be evident in *Apocryptodon punctatus*. In addition, the strong and warm Kuroshio Current, which hinders dispersion of fishes from north to south (Matsuura and Senou 2012, Kuriiwa et al. 2014), flows northward off the coast of southern Miyazaki, and no population of *Apocryptodon punctatus* has been found near Kushima City until now. Therefore, it is reasonable to conclude that the population of *Apocryptodon punctatus* from an estuary of Kushima City reproduces locally, and is isolated from other populations in Japanese waters.

Miyazaki Prefecture has lost large areas of estuarine tidal flats since the 1980’s mainly due to the restriction of Hitotsuba lagoon and the development of Miyazaki Port near Miyazaki City (Miura et al. 2005, Miura 2008). It is possible that the preferable estuarine habitat for *Apocryptodon punctatus* has been lost in the prefecture except for the estuary of Kushima City. Fishes are a good indicator of estuarine ecosystem health, and are useful to assess and monitor anthropogenic impacts (Whitfield and Elliott 2002, Harrison and Whitfield 2004). Species such as *Apocryptodon punctatus*, which has a relatively sparse distribution and specificity for habitat and a symbiotic partner, can be used as indicator species for environmental monitoring in estuaries by coupling their occurrence with biodiversity and functional parameters (e.g. biomass, water quality, etc). That aside, it is important that the threatened status of this and other species is recognized and that preferable habitat is maintained. This is all the more important when a population is isolated, such as the present case. Urgent action is required to conserve this population, with the first step being to record Kushima City (Miyazaki Prefecture) in the Red Data Book as the southernmost limit for *Apocryptodon punctatus* in Japanese waters.

**Other materials.** Catalogue number and collection data of additional museum specimens for distributional records of *Apocryptodon punctatus* in Fig. 2 and Table 2 are as follows (all the specimens collected by Ryutei Inui and his colleagues, specimen size expressed in SL)—Tokushima Prefecture: TKPM-P 23222 (1, 47.4 mm, Anan City, 16 Apr. 2011); Hyogo Prefecture: TKPM-P 24521 (1, 27.7 mm, Tatsuno City, 17 Nov. 2011); Okayama Prefecture: TKPM-P 24549 (1, 49.5 mm, Okayama City, 29 Apr. 2011), TKPM-P 24550 (3, 37.7–43.4 mm, Okayama City, 16 Nov. 2011), TKPM-P 24551 (1, 52.2 mm, Kurashiki City, 2 June 2011); Yamaguchi Prefecture: TKPM-P 24637 (2, 48.7–50.3 mm, Shimonoseki City, 27 July 2011), TKPM-P 24638 (2, 25.7–40.7 mm, Sanyo-onoda City, 8 Aug. 2010), TKPM-P 24639 (1, 43.3 mm, Sanyo-onoda City, 27 July 2011), TKPM-P 24640 (1, 43.5 mm, Ube City, 27 July 2011), TKPM-P 24641 (1, 44.5 mm, Yamaguchi City, 28 July 2011), TKPM-P 24642 (1, 40.7 mm, Yamaguchi City, 26 Apr. 2009); Kagawa Prefecture: TKPM-P 23514 (1, 21.3 mm, Marugame City, 6 Oct. 2011); Ehime Prefecture: TKPM-P 23567 (1, 25.3 mm, Ainan-cho Town, 12 Oct. 2011), TKPM-P 23568 (1, 35.7 mm, Saijyo City, 15 July 2011), TKPM-P 23569 (1, 16.5 mm, Saijyo City, 20 Sep. 2008); Fukuoka Prefecture: TKPM-P 25279 (3, 17.9–55.0 mm, Kitakyushu City, 11 Aug. 2011), TKPM-P 25280 (4, 17.4–18.3 mm, Kitakyushu City, 11 Aug. 2011), TKPM-P 25281 (1, 16.7 mm, Yukuhashi City, 11 Aug. 2011), TKPM-P 25282 (1, 16.9 mm, Yukuhashi City, 11 Aug. 2011), TKPM-P 25283 (42.9 mm, Yukuhashi City, 8 June 2006), TKPM-P 25284 (1, 48.6 mm, Kitakyushu City, 24 Mar. 2008); Oita Prefecture: TKPM-P 25068 (1, 58.5 mm, Nakatsu City, 12 Aug. 2011).
